# How to predict early clinical outcomes and evaluate the quality of primary total knee arthroplasty: a new scoring system based on lower-extremity angles of alignment

**DOI:** 10.1186/s12891-020-03528-3

**Published:** 2020-08-03

**Authors:** Ziming Chen, Zhantao Deng, Qingtian Li, Junfeng Chen, Yuanchen Ma, Qiujian Zheng

**Affiliations:** 1 Department of Orthopedics, Guangdong Provincial People’s Hospital, Guangdong Academy of Medical Sciences, Guangzhou City, 510080 Guangdong Province China; 2 Centre for Orthopaedic Translational Research, Medical School, University of Western Australia, Nedlands, Australia

**Keywords:** Total-knee arthroplasty, Predictors, Scoring system, Clinical outcome, Alignment, Grade approach

## Abstract

**Background:**

A method that can accurately predict the outcome of surgery can give patients timely feedback. In addition, to some extent, an objective evaluation method can help the surgeon quickly summarize the patient’s surgical experience and lessen dependence on the long wait for follow-up results. However, there was still no precise tool to predict clinical outcomes of total knee arthroplasty (TKA). This study aimed to develop a scoring system to predict clinical results of TKA and then grade the quality of TKA.

**Methods:**

We retrospectively reviewed 98 primary TKAs performed between April 2013 and March 2017 to determine predictors of clinical outcomes among lower-extremity angles of alignment. Applying multivariable linear-regression analysis, we built Models (i) and (ii) to predict detailed clinical outcomes which were evaluated using the Knee Society Score (KSS). Multivariable logistic-regression analysis was used to establish Model (iii) to predict probability of getting a good clinical outcome (PGGCO) which was evaluated by Knee Injury and Osteoarthritis Outcome Score (KOOS) score. Finally, we designed a new scoring system consisting of 3 prediction models and presented a method of grading TKA quality. Thirty primary TKAs between April and December 2017 were enrolled for external validation.

**Results:**

We set up a scoring system consisting of 3 models. The interpretations of Model (i) and (ii) were good (R^2^ = 0.756 and 0.764, respectively). Model (iii) displayed good discrimination, with an area under the curve (AUC) of 0.936, and good calibration according to the calibration curve. Quality of surgery was stratified as follows: “A” = PGGCO ≥0.8, “B” = PGGCO ≤0.6 but < 0.8, and “C” = PGGCO < 0.6. The scoring system performed well in external validation.

**Conclusions:**

This study first developed a validated, evidence-based scoring system based on lower-extremity angles of alignment to predict early clinical outcomes and to objectively evaluate the quality of TKA.

## Introduction

Total knee arthroplasty (TKA) has long been widely used to treat knee joint diseases. However, 15–30% of patients are dissatisfied with TKA clinical outcomes [1–3]. After TKA, some patients present with various persistent complications, such as pain, ankylosis, and joint clicking [3, 4]. Unfortunately, the primary reason for such problems remains unclear. Previous studies have demonstrated that an appropriate angle of implantation plays a pivotal role in clinical efficacy [5–9]. In the previous studies, the hip–knee–ankle angle (HKA) of the lower extremity within 3° and tibial and femoral implants perpendicular to their respective anatomical axes on the sagittal plane were considered the determining factors of surgical success [10, 11].

Nevertheless, numerous recent studies have questioned the importance of HKA in restoring the affected limb’s function after TKA [5, 12–18]. Many other lower-extremity angles of alignment have also been studied for their correlations with clinical outcomes. Some are part of HKA, such as mechanical medial proximal tibial angle (mMPTA) and coronal femoral angle (CFA); there are also others, such as joint line orientation angle (JLOA) and distal femoral valgus resection (DFVR) [18, 19]. However, there is no consensus on which of these angles correlate to clinical outcome in TKA [5, 12, 20–22]. Thus, correlations between angles of limb alignments and clinical outcomes should be further clarified.

In addition, functional scores obtained during follow-up are often necessary to evaluate TKA quality [13, 18]. This makes research difficult because follow-up must take a long time, and there is a possibility of losing data during this period. At present, the criterion of HKA neutrality in post-operative radiographs is often used to evaluate surgical quality, but it is controversial and is not validated, as there is no precise tool by which to predict clinical outcomes in TKA. For the clinical outcomes of TKA, the early outcomes are always the remarkable and important events for surgeons and in researches, and represent the quick recovery and the preliminary surgery effect [23, 24]. Thus, a precise prediction model for early clinical outcomes will be useful on various occasions.

This study aimed to identify relative predictors of early clinical outcomes in primary TKA among lower-extremity angles of alignment and to develop a precise, practical, convenient, detailed, and valid scoring system to predict the clinical results of and then grade the quality of TKA.

## Materials and methods

This study was performed in accordance with the ethical standards laid down in the 1964 Declaration of Helsinki. All of the patients gave their informed consent prior to their inclusion in the study. The study protocol was approved by an institutional review board (name of institution blinded).

### Source of data and participants

**Fig. 1:**
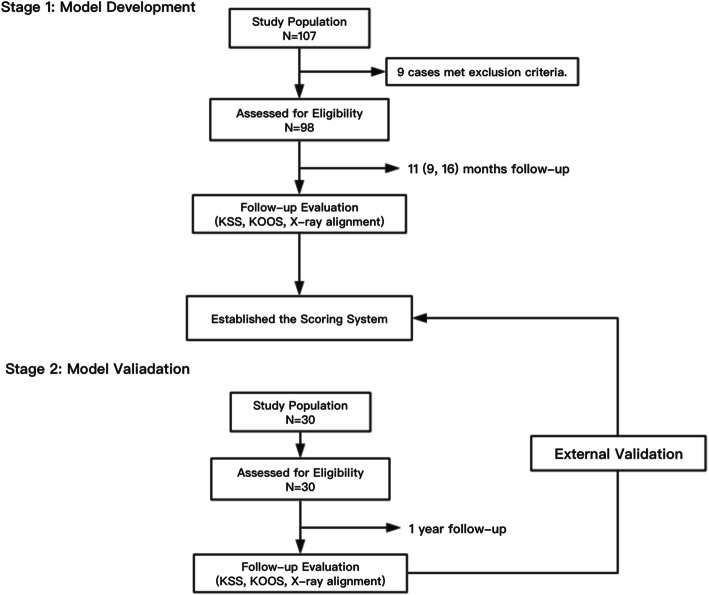
Study design flowchart. N: number of cases

This study was a single-center, participant- and outcome assessor–blinded, ambispective cohort study. In the first stage, we retrospectively included patients who underwent primary TKA in the Department of Orthopedics (name of institution blinded) between April 2013 and March 2017 in the modeling group. The second stage of research consisted of a prospective study analyzing patients who underwent primary TKA at the same institution between April and December 2017, conducted to evaluate the model built with data from the first group of patients. A flowchart of the study design is shown in Fig. 1.

### Inclusion and exclusion criteria

Inclusion criteria were as follows: patients age > 18 years, patients with osteoarthritis or rheumatoid arthritis, and patients with complete imaging data from frontal and lateral knee X-rays and coronal standing full-length radiographs. Exclusion criteria were as follows: patients with medical history of ipsilateral femoral lesions, knee joint fracture, or neuromuscular disease that affected the knee joint function; and patients implanted with a highly constrained prosthesis.

### Clinical data

We recorded baseline characteristics, including age, sex, follow-up time, type of deformity (varus or valgus), and side of the operative extremity. Radiological outcomes, including DFVR, sagittal tibial angle (STA), sagittal femoral angle (SFA), CFA, mechanical lateral distal femoral angle (mLDFA), mMPTA, and JLOA, were measured on post-operative X-ray images. DFVR, CFA, mLDFA, mMPTA, and JLOA were measured on standing full-length radiographs, while STA and SFA were measured on lateral radiographs.

**Fig. 2:**
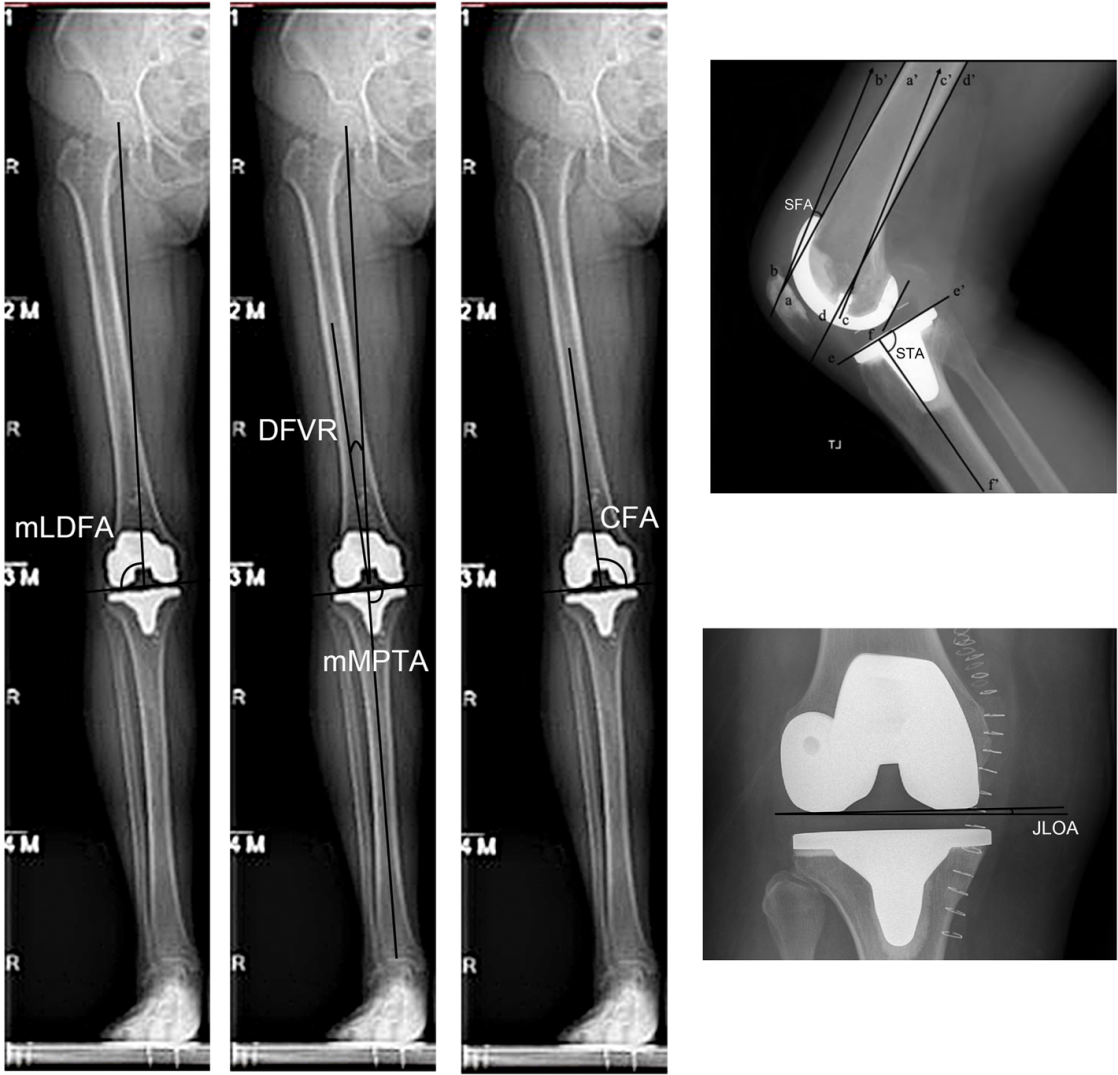
Radiological measurements. DFVR: distal femoral valgus resection. mMPTA: mechanical medial proximal tibial angle. mLDFA: mechanical lateral distal femoral angle. CFA: coronal femoral angle. SFA: sagittal femoral angle. STA: sagittal tibial angle. JLOA: joint line orientation angle

Angular measurements were made as follows using Kodak Carestream version 10.2 picture archiving and communication system (PACS) software (Carestream Health, Inc., Rochester, New York, US, 2008; Fig. 2).

Mechanical femoral axis (MFA) referred to the line connecting the center of the femoral head, as determined by a best-fit circle, and the midpoint of the widest dimension of the distal femur. Mechanical tibial axis (MTA) was defined as the line connecting the center of the tibial spines to the center of the talus. Anatomical femoral axis (AFA) referred to the line connecting the midpoint of the endosteal cortices of the femoral isthmus to the midpoint of the femur 10 cm proximal to the joint line. Similarly, anatomical tibial axis (ATA) was defined as the line connecting the midpoint of the tibia’s midshaft to the midpoint of the tibia 10 cm distal to the joint line [25, 26]. DFVR was defined as the angle between the MFA and the AFA [27]. mMPTA was defined as the medial angle between the MTA and the proximal tibial joint line, while mLDFA referred to the lateral angle between the MFA and the distal femoral joint line [28]. STA referred to the angle between the ATA and the joint line on lateral images. SFA referred to the angle between the AFA and the longitudinal axis of the femoral implant on lateral images. JLOA was the angle between the joint line and the horizontal line. CFA represented the medial angle between the AFA and the distal femoral joint line [18, 19].

Two independent observers performed radiographic measurements, the first one doing so twice, and then an inter-observer correlation was calculated. The data measured the first time by the first observer were used in the next statistical analysis.

At follow-up, each subject was asked to answer the Objective Knee Score and Functional Score subscales of the Knee Society Score (KSS), as well as a modified version of the Knee Injury and Osteoarthritis Outcome Score (KOOS) that did not include the Sports and Recreation subscale. The overall clinical outcome was evaluated by a composite score that was calculated as the average of KOOS subscale scores. The KSS questionnaire was validated and responsive for assessing objective clinical outcomes after TKA [29]. According to the user manual, the objective knee score of KSS includes a visual analog scale score of pain, as well as an evaluation of alignment, ligament stability, and range of motion, along with deductions for flexion contracture or extensor lag. Compared with KOOS and other scales, it is better targeted to patients who have undergone TKA; therefore, we used it in the current study for detailed scoring. However, The Knee Society has declared that KSS should be reported only as separate scores rather than as an overall single score with statistical validity [30]. The KOOS is an instrument designed to assess the patient’s opinions about their knee and associated problems, and its psychometric properties have been assessed in several studies from all over the world [31, 32]. It permits creation of a composite score by calculating an average from its subscale scores, which ensures similar weight from all of the subscales in the composite score [33, 34]. The aim of this study was to establish an objective scoring system for clinical outcome in TKA, so we did not include the Patient Expectations and Satisfaction subscale of the KSS during follow-up. Most patients involved in the study mentioned that they avoided activities such as squatting, running, jumping and kneeling for several reasons, such as advanced age, co-existing diseases, and contralateral knee pain. Therefore, we did not include the KOOS’s Sports and Recreation subscale [20]. Mugnai R et al. stratified patients by KOOS score as “excellent” (score ≥ 80 points), “mild” (score < 80 and ≥ 60), and “poor” (score < 60) [20]. In order to establish a practical and convenient scoring system, we dichotomized patients as either “good” (score ≥ 70 points) or “poor” (score < 70), according to their composite KOOS scores, as the composite comment. In addition, in our study, unity of results for the KSS and KOOS was tested.

### Outcome

The major result of this study was to establish a new scoring system based on lower-extremity angles of alignment in order to predict early outcomes in TKA, and then to evaluate the quality of surgery. We chose 3 prediction models for the scoring system. Models (i) and (ii) were used to predict Objective Knee Score and Functional Score, respectively, for the Detailed Scoring part of the system. Model (iii) was used to predict overall comment for the Overall Scoring part of the system. The overall comment was described as the probability of getting a good clinical outcome (PGGCO). Quality of surgery was graded as follows: “A,” PGGCO ≥0.8; “B,” 0.6 ≤ PGGCO < 0.8; and “C,” PGGCO < 0.6.

### Validation of the scoring system

We prospectively performed external validation on separate patients from the same institution, who were not included in our established models. Follow-up time was designed as 12 months.

### Sample size

No formal power calculation was performed to determine sample size for the regression analysis. The sample size was adequate to satisfy the recommended guide of 10 events per predictor [35]. Thus, with up to 7 initial predictors, 70 patients were sufficient to establish a regression model. In order to establish a more stable and accurate scoring system, we enrolled 98 cases to establish models and 30 cases for external validation.

### Statistical analysis

We used the Kolmogorov–Smirnov test to evaluate whether measurement data were in accordance with the normal distribution. Non-normally distributed data were expressed as median (25th and 75th percentiles), while normally distributed data were expressed as mean ± standard deviation (SD). We used an intra-class correlation coefficient (ICC) to evaluate intra- and inter-observer correlations of measurements, and we assessed correlations between measurement data using Pearson’s correlation coefficient (PCC). Using previously described semiquantitative criteria, we graded correlation coefficients as follows: excellent, 0.9 ≤ *r* ≤ 1; good, 0.7 ≤ *r* ≤ 0.89; moderate, 0.5 ≤ *r* ≤ 0.69; low, 0.25 ≤ *r* ≤ 0.49; and poor, *r* ≤ 0.24 [36].

All of the radiological outcomes were applied as initial potential predictors to develop the prediction models for clinical outcomes [37, 38]. We used the Kaiser–Meyer–Olkin (KMO) and Bartlett’s test as well as correlation analysis among variables to evaluate the multicollinearity of independent variables in order to select suitable potential predictors.

We performed multiple linear-regression analyses with backward stepwise selection (*P* = 0.05) to build Model (i), quantifying the relationship between predictors and KSS Objective Knee Score; as well as to build Model (ii), quantifying the relationship between predictors and KSS Functional Score. Before performing multiple logistic-regression analyses, we conducted univariable logistic-regression analyses between all selected potential predictor variables and the composite KOOS score to select final predictor variables with *P* < 0.05. Then we used multivariable logistic-regression analysis to build Model (iii), quantifying the relationship between predictors and the composite comment. The residuals and data requirements for linear relation, normality, and equality of variance were tested.

We used goodness of fit to assess the predictive performance of Models (i) and (ii), while that of Model (iii) was assessed by calibration and discrimination. For Models (i) and (ii), goodness of fit was assessed by *R*^2^ value. We used an analysis of variance (ANOVA) analysis to test whether the model (i) and (ii) had statistical significance, and used Omnibus analysis to test whether the model (iii) had statistical significance. For Model (iii), we assessed calibration using a calibration curve [39]. A slope on the 45° line represented perfect calibration. We also performed a Hosmer–Lemeshow goodness-of-fit test as the supplement [40]. *P* ≥ 0.05 indicated goodness of fit. Discrimination was typically characterized using the area under the curve (AUC) with 95% confidence intervals (CI) of the receiver operating characteristic (ROC) curve. An AUC of 0.5 indicated no discrimination, while an AUC of 1.0 meant perfect discrimination [41]. For external validation, we calculated the observe/expect (O/E) ratio by dividing the mean of the actual score by that of the predictive score in the validation group.

We used R software version 3.5.2 (Ihaka and Gentleman, 2018) for logistic regression modeling and SPSS software version 23 (SSPS, Inc., Chicago, Illinois, US) for linear regression modeling and descriptive analyses. Significance for all of the tests was defined as *P* < 0.05.

## Results

We included 98 primary TKAs (67 patients) between April 2013 and March 2017 to develop our models. Baseline characteristics and clinical outcomes are shown in Table 1. No severe post-operative complications requiring revision TKA were observed during the subsequent follow-up, and no data is missing in this study.

**Table 1:** Baseline characteristics and clinical outcomes

	Modeling Group	Validation Group
**Baseline Characteristics**		
N	98	30
Gender		
Male (n, %)	8, 8%	5, 17%
Female (n, %)	90, 92%	25, 83%
Age (years)^b^	66.28 ± 9.61	66.23 ± 8.52
Follow-up time (months)^a^	11 (9, 16)	12
Type of deformity		
Varus (n, %)	74, 76%	23, 77%
Valgus (n, %)	24, 24%	7, 23%
Side of operative extremity		
Left (n, %)	45, 46%	15, 50%
Right (n, %)	53, 54%	15, 50%
**Clinical Outcomes**		
Objective knee score^b^	86.91 ± 3.39	85.30 ± 3.82
Functional score^b^	82.73 ± 2.89	80.57 ± 3.08
Composite KOOS score^b^	74.34 ± 3.50	75.93 ± 7.80
Overall comment		
Good clinical outcome (N, %)	82, 84%	22, 73%
Bad clinical outcome (N, %)	16, 16%	8, 27%

^a^Data are non-normally distributed and expressed as M (P25, P75). ^b^Data are normally distributed and expressed as *x̄* ± SD. N: total number of cases. n: subset of cases. *KOOS* Knee Injury and Osteoarthritis Outcome Score, *M* median. P25: 25th percentile. P75: 75th percentile. *x̄*: mean. *SD* standard deviation

Inter-observer correlations for radiological outcomes were excellent (ICC > 0.97; Table 2), as were intra-observer correlations (ICC > 0.98). These data indicated that the measurement method in this study was highly repeatable and accurate. The correlation between detailed score and composite KOOS score is shown in Table 3; it indicated eligible unity between the Detailed Scoring and Overall Scoring parts of the scoring system.

**Table 2:** Inter-observer correlations for post-operative radiological outcomes

Radiological Outcomes	Observer A	Observer B	ICC (95% CI)	***P***-value
DFVR (°)^a^	5.61 (4.05, 7.37)	5.74 (4.13, 7.59)	0.998 (0.997–0.999)	*P* < 0.001
STA (°)^b^	87.74 ± 3.52	87.32 ± 3.61	0.992 (0.989–0.995)	*P* < 0.001
SFA (°)^a^	2.30 (1.33, 4.61)	2.55 (1.43, 5.02)	0.970 (0.955–0.980)	*P* < 0.001
CFA (°)^b^	95.24 ± 2.76	95.62 ± 2.76	0.994 (0.991–0.996)	*P* < 0.001
mMPTA (°)^b^	89.34 ± 2.65	89.33 ± 2.87	0.983 (0.975–0.989)	*P* < 0.001
mLDFA (°)^b^	91.37 ± 3.04	90.94 ± 3.08	0.997 (0.995–0.998)	*P* < 0.001
JLOA (°)^b^	2.93 ± 2.14	2.57 ± 2.11	0.990 (0.984–0.993)	*P* < 0.001

^a^Data are non-normally distributed and expressed as M (P25, P75). ^b^Data are normally distributed and expressed as $\overline{x}$± SD. M: median. P25: 25th percentile, P75: 75th percentile. *x̄*: mean. *SD* standard deviation, *ICC* intra-class correlation coefficient, *CI* confidence interval, *DFVR* distal femoral valgus resection, *mMPTA* mechanical medial proximal tibial angle, *mLDFA* mechanical lateral distal femoral angle, *CFA* coronal femoral angle, *SFA* sagittal femoral angle, *STA* sagittal tibial angle, *JLOA* joint line orientation angle

**Table 3:** Correlation between detailed score and composite KOOS score

	Objective Knee Score	Functional Score	Composite KOOS Score
*x̄* ± SD	86.91 ± 3.39	82.73 ± 2.89	74.34 ± 3.50
*r*	0.856	0.829	
*P*-value	<0.001	<0.001	

*x̄*: mean. *SD* standard deviation. *r*: Pearson correlation coefficient. *KOOS* Knee Injury and Osteoarthritis Outcome Score

Correlation analysis of different lower-extremity angles of alignment revealed certain correlations between mLDFA and JLOA (*r* = 0.50; *P* < 0.001), mLDFA and DFVR (*r* = 0.63; *P* < 0.001), and mLDFA and CFA (*r* = − 0.366; *P* < 0.001). After elimination of mLDFA, there was no collinearity or very weak collinearity among variables. The KMO value was 0.40, also indicating no significant or very weak collinearity among independent variables. Thus, DFVR, STA, SFA, CFA, mMPTA, and JLOA were ultimately selected as potential predictors for development of our models. The residuals and the data requirements for linear relation, normality, and equality of variance in each model were satisfied.

### Development of the scoring system

**Fig. 3:**
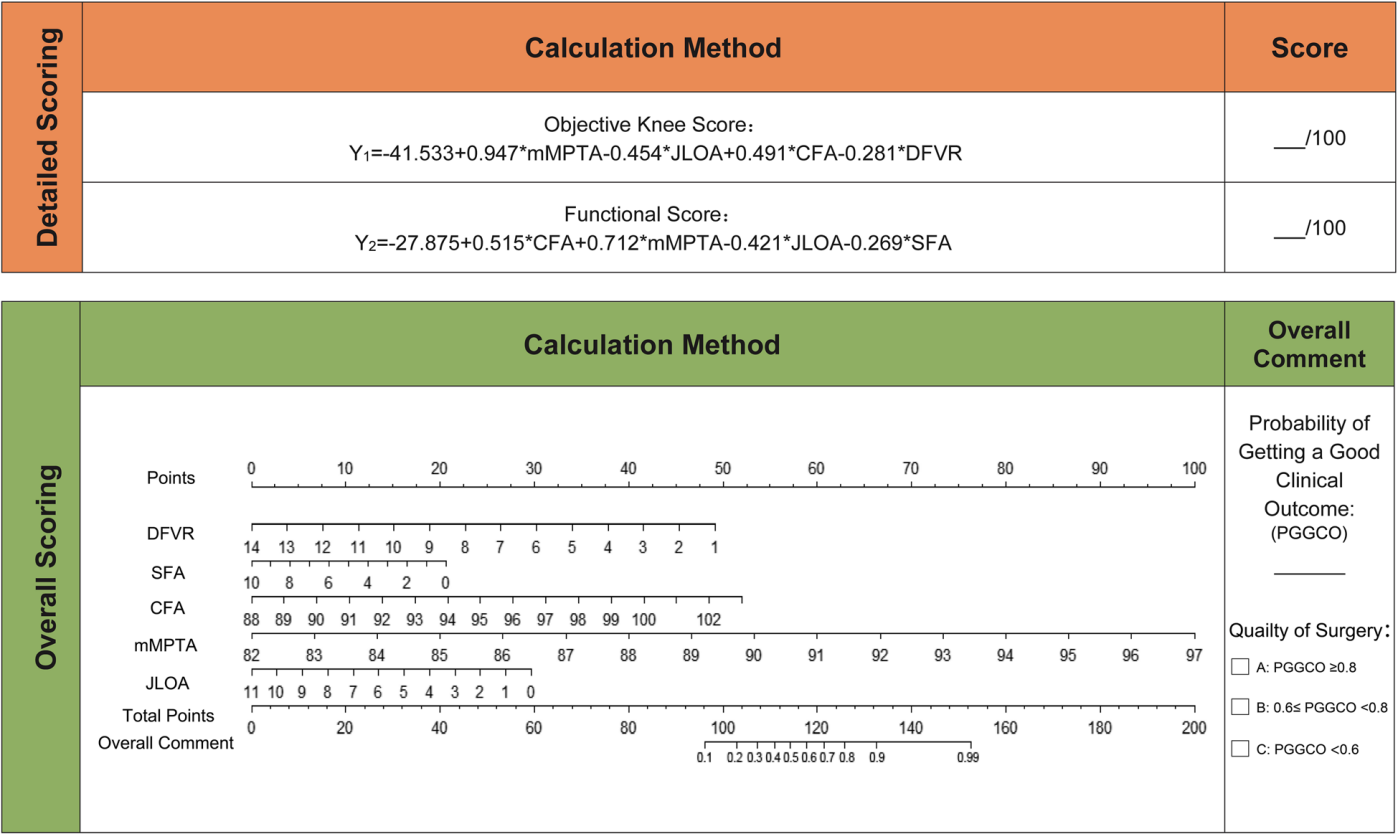
A new scoring system based on lower-extremity angles of alignment for TKA clinical outcome and quality. TKA: total-knee arthroplasty. DFVR: distal femoral valgus resection. mMPTA: mechanical medial proximal tibial angle. CFA: coronal femoral angle. SFA: sagittal femoral angle. STA: sagittal tibial angle. JLOA: joint line orientation angle

The new scoring system consisted of 2 parts and 3 models (Fig. 3).

### Objective knee score in detailed scoring part

After multiple linear-regression analyses with backward stepwise selection, we selected mMPTA, JLOA, CFA, and DFVR as predictors for Objective Knee Score. The equation for Model (i) was as follows:

$$ {Y}_1=-41.533+0.947\kern0.5em \ast \kern0.5em \mathrm{mMPTA}-0.454\kern0.5em \ast \kern0.5em \mathrm{JLOA}\kern0.5em +\kern0.5em 0.491\kern0.5em \ast \kern0.5em \mathrm{CFA}\kern0.5em \hbox{-} \kern0.5em 0.281\kern0.5em \ast \kern0.5em \mathrm{DFVR}. $$

The *R*^2^ value of Model (i) was 0.756, indicating that the interpretation of this model was good. ANOVA analysis showed that the *F*-value was 72.09 and *P* < 0.001, indicating that Model (i) had statistical significance.

### Functional score in detailed scoring part

After multiple linear-regression analyses with backward stepwise selection, we selected mMPTA, JLOA, CFA, and SFA as predictors for Functional Score. The equation for Model (ii) was as follows:

$$ {Y}_2=-27.875\kern0.5em +\kern0.5em 0.515\kern0.5em \ast \kern0.5em \mathrm{CFA}\kern0.5em +\kern0.5em 0.712\kern0.5em \ast \kern0.5em \mathrm{mMPTA}\kern0.5em -\kern0.5em 0.421\kern0.5em \ast \kern0.5em \mathrm{JLOA}\kern0.5em -\kern0.5em 0.269\kern0.5em \ast \kern0.5em \mathrm{SFA}. $$

The *R*^2^ value of Model (ii) was 0.764, indicating that the interpretation of this model was good. ANOVA analysis showed that the *F*-value was 75.367 and *P* < 0.001, indicating that Model (ii) had statistical significance.

### Overall scoring part

After performing univariable logistic-regression analyses, we selected DFVR, SFA, CFA, mMPTA, and JLOA as predictors for overall score. The equation for Model (iii) was as follows:

$$ \mathrm{logit}(p)=\kern0.5em -103.69\kern0.5em -\kern0.5em 0.454\kern0.5em \ast \kern0.5em \mathrm{DFVR}-0.248\kern0.5em \ast \kern0.5em \mathrm{SFA}+0.417\kern0.5em \ast \kern0.5em \mathrm{CFA}\kern0.5em +\kern0.5em 0.801\kern0.5em \ast \kern0.5em \mathrm{mMPTA}\kern0.5em -0.324\kern0.5em \ast \kern0.5em \mathrm{JLOA} $$

**Fig. 4:**
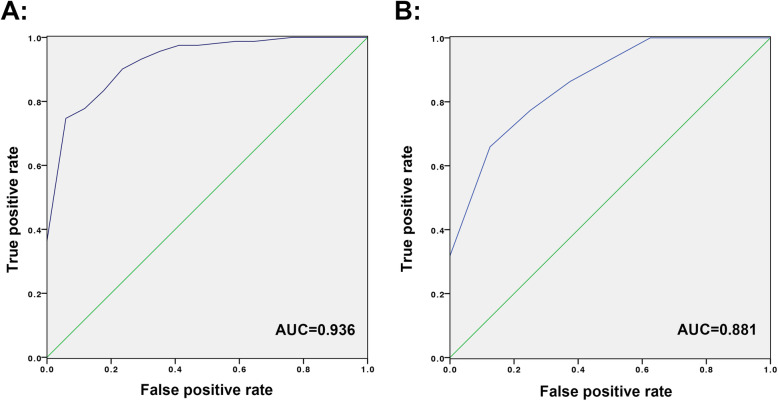
ROC curve for Model (iii). **a** ROC curve of Model (iii) in modeling group. **b** ROC curve of Model (iii) in validation group. AUC: area under the curve. ROC: receiver operating characteristic

**Fig. 5:**
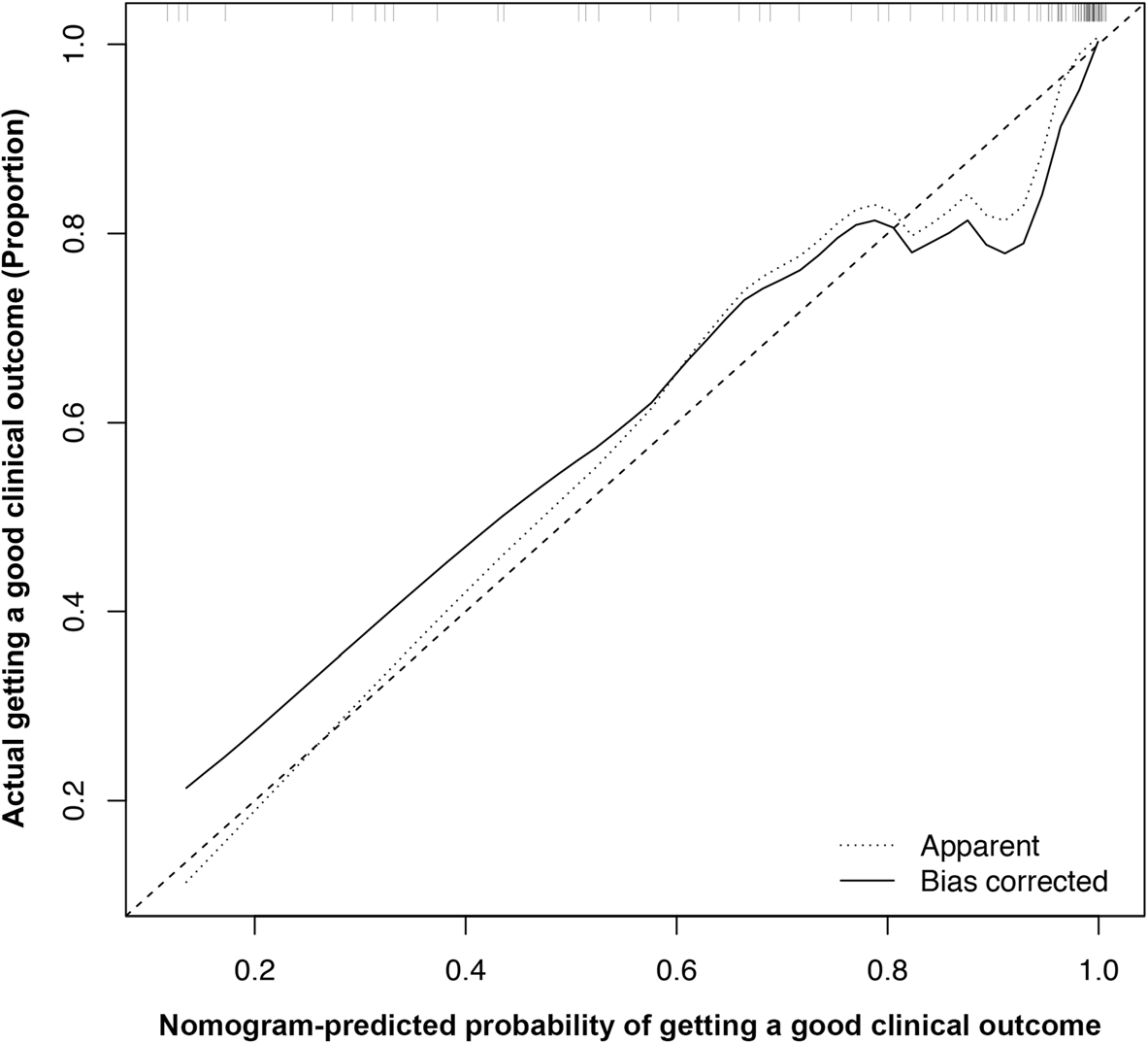
Calibration curves of Model (iii): good-clinical-outcome nomogram prediction in the modeling group. *X*-axis: predicted probability of getting a good clinical outcome (PPGCO). *Y*-axis: actual getting a good clinical outcome. Diagonal dotted line: a perfect prediction by an ideal model. Solid line: performance of the nomogram; the closer the fit to the diagonal dotted line, the better the prediction

The ROC curve is shown in Fig. 4a. The AUC value was 0.936 (95% CI, 0.910–0.962) > 0.75, indicating that the discrimination of Model (iii) was good [40]. The calibration curve is shown in Fig. 5. The Hosmer–Lemeshow goodness-of-fit test showed *P* = 0.572, which meant calibration was good. Omnibus analysis showed that *P* < 0.001, indicating that Model (iii) had statistical significance. Model (iii) was presented as a nomogram in the final scoring system (Fig. 3).

### External validation

We enrolled 30 primary TKAs (18 patients) between April and December 2017 for validation. Baseline characteristics and clinical outcomes are presented in Table 1.

In Model (i), 27/30 (90%) outcomes were predicted correctly by the 95% CI of the predictive value; O/E value was 0.982. In Model (ii), 26/30 (87%) were predicted correctly by the 95% CI of the predictive value; O/E value was 0.979. The ROC curve for Model (iii) is shown in Fig. 4b. The AUC value was 0.881 (95% CI, 0.816–0.946) > 0.75, indicating that the discrimination of Model (iii) was good [40]. Surgical quality in the validation group was graded as followed: 20 cases as “A,” 6 cases as “B,” and 4 cases as “C.” We observed 18 cases in A (90%), 4 cases in B (66.67%), and 0 cases in C (0%) to have obtained good clinical outcomes that met the definition of the grade in question.

## Discussion

This study ascertained which lower-extremity angles of alignment were relevant to clinical outcomes in primary TKA. We found that mMPTA, JLOA, CFA, and DFVR were relevant to Objective Knee Score, while mMPTA, JLOA, CFA, and SFA were relevant to Functional Score. In addition, DFVR, SFA, CFA, mMPTA, and JLOA correlated with overall score. Next, we established a new scoring system to predict early clinical outcomes in TKA, and we tested and verified the feasibility of this system. Finally, we proposed and validated an objective method to evaluate quality of surgery. This study offered a possibility to select the patients in high risk of poor clinical outcomes. And for these high-risk patients, advanced exercise rehabilitation can be provided corresponding to the score of Detailed Scoring part. For example, for patients with low functional score, the role of proper walking will be emphasized to patients and patients will be encouraged to spend more time walking. With low objective knee score, patients will be asked for more passive training of knee and muscle training designed to strengthen, promote healing and increase the range of motion of the knee. A randomized controlled trial to evaluate the effect of providing certain rehabilitation to high-risk patients selected by our system is ongoing.

STA, SFA, mMPTA, and mLDFA belong to the angles of component alignment, while mMPTA and mLDFA determine the coronal alignments of the tibial and femoral components on the tibia and femur, respectively. Many studies [11, 42, 43] have proven that mMPTA and mLDFA were related to the post-operative function of the knee; our results were similar. In addition, we found that sagittal-plane component alignment, including SFA and STA, was weakly or not at all correlated with surgical outcome, similar to the findings of Antony J et al. [21] DFVR was an individual anatomical characteristic and was not changed in TKA. However, DFVR combining with CFA determines mLDFA, as “mLDFA = 180° − CFA + DFVR.” Collinearity among these angles was also proven in this study. Thus, by replacing mLDFA with DFVR and CFA, a more accurate prediction model can be obtained. JLOA mainly determines the alignment of the prosthesis and its position relative to space. The true anatomy of the femur and tibia allows the joint line to be parallel to the ground during the normal-stance phase of gait [44]. Ji HM et al. found that in comparison with mechanically aligned TKA, kinematically aligned TKA can align the knee joint line horizontally, a fact often discussed in studies of kinematically aligned TKA’s efficacy [14, 18, 45]. This study confirmed that JLOA is a factor affecting clinical outcomes in TKA.

A method that can accurately predict the outcome of surgery can give patients timely feedback, allowing them to form appropriate expectations of post-operative clinical outcomes. In addition, to some extent, an objective evaluation method can help the surgeon quickly summarize the patient’s predicted surgical experience and lessen dependence on the long wait for follow-up results. Gwo-Chin L et al. tried to use the surgeon’s subjective view of the technical quality of surgery to predict post-operative function, but ultimately it did not work unless the quality score was extremely low [46]. This study successfully used post-operative radiological outcomes as objective data to predict clinical outcomes, and our prediction method has been verified. To the best our knowledge, the present study is the first prediction system based on several postoperative lower-extremity angles of alignment to predict objective clinical outcomes. Many other score systems predicting clinical outcomes focused on patients’ satisfactory instead of the objective outcomes, and others did not include all radiographic alignment features [47, 48]. The present study provides a new way of thinking to evaluate the quality of total knee arthroplasty. Compared with other score systems which could predict the objective outcomes, the accuracy of the present scoring system is better [49, 50].

Postoperative angles of alignments were confirmed to be objectively important in the present study, but the preoperative lower extremity alignments state might also affect the 1-year clinical outcomes. Thus, an analysis using reduced angles as the dependent variables in the models instead of post-operative angles was conducted (data not shown). However, results showed that there were no model built with statistical significance. We considered that it might can be an evidence to support mechanical alignment theory in terms of 1-year outcomes. Described by Insall et al., the mechanical alignment is the widest method used in TKA probably due to the high reproducibility and easiness [51]. In mechanical alignment theory, postoperative angle is the most important parameter for TKA outcomes. According to some systematic literature reviews, a neutral postoperative mechanical axis remains the optimal guide to alignment, regardless of the preoperative condition [52, 53]. However, this conclusion is still controversial. For example, kinematic alignment theory is another popular hypothesis [54–56].

Apart from alignment, the surgical efficacy of TKA is affected by various factors, including rotation angle of the implant, soft-tissue balance status, and patellofemoral tracking, subjective indicators such as education level of patients, etc. [5, 57–59] Our scoring system focuses only on alignment, mainly because other factors like soft-tissue balance status and patellofemoral tracking are difficult to evaluate objectively. Besides, tomography (CT) is not a routine examination after TKA in many medical centers but it is necessary for measuring the rotation angle of the implant. To encourage widespread use of this scoring system, this study did not include the rotation angle of the prosthesis. Furthermore, the patient expectations and satisfaction are also essential to be considered as postoperative outcomes for TKA. We tried to use the patient expectations and satisfaction score of KSS as the clinical outcomes, however the results showed there were no model built with statistical significance based on our existing independent variables (data not shown). We considered there might be more factors affected subjective outcomes, such as preoperative pain and disability, education level, telerehabilitation, age, and so forth [60–62]. Thus, a further prediction model including these factors as independent variables to predict patient expectations and satisfaction score can be more integrated and comprehensive.

Several limitations of this study should be acknowledged. First, as with 2-dimensional radiographic assessments, limb rotation can affect the measurements. However, all 2-dimensional images following a detailed, uniform protocol in order to minimize this bias in the present study. Moreover, although the measurement methods in this study were standard and consistent with previous studies, their accuracy has been questioned. For example, femoral neck–shaft angle may affect the mechanical axis of the femur [28, 63]. A more reliable method is to use CT results. However, it will increase the economic burden for patients, as well as CT is not a routine examination after TKA in many clinical centers. Thus, further study to perform the present scoring system measuring alignments by CT images and compared the efficacy with measuring by x-ray images will be helpful. Second, the radiological outcomes in this study came from a single observer, which may have led to bias. However, according to our ICC, the results from a single operator was highly reliable and repeatable, which was similar to previous studies [64, 65]. Thus, in the present study, finally, the radiological outcomes were measured by one operator like some previous studies [52, 66]. Third, this study focused only on early clinical effects. Jeffrey J et al. demonstrated that inappropriate joint alignment could lead to increased implant stress, poor patient outcomes, and decreased rates of survival [43]. A prediction system for early clinical outcomes is useful, but it will be more informative if it can predict mid- to long- term outcomes. Thus, for our further study, we plan to focus on long-term clinical outcomes and survival rates additionally. Fourth, as a single-center research, surgeons’ methods to balance soft-tissue and adjust patellofemoral track were basically similar, which reduced the effect of confounding factors on our scoring system to a certain extent. Thus, even if the sample size of this study met minimum requirements and all *p*-values of the models were less than 0.001, further multi-center and large-sample studies to adjust and evaluate the models are necessary. The aim of further studies is to ensure that the scoring system can work accurately under the influence of confounding factors, as well as to popularize it and thus encourage its adoption by other clinical centers. Fifth, as the prevalence of TKA was higher among women than among men, in the present study females constituted a majority of patients [67]. However, according to a meta-review including 32 studies and almost 30,000 patients, the overall effect of gender was small and of minimal clinical importance [68]. Finally, some patients with bilateral TKA were considered to be two independent TKAs in our study, which potentially controlled the patient’s baseline characteristics. Moreover, because full-length X-ray was not the routine post-operative examination item in our hospital especially before 2017, most of the patients who underwent full-length X-ray were patients with poor preoperative knee function or long hospitalization time, which also potentially controlled the preoperative knee function. All these biases might lead to a high fitness of the model and it might be the reason why the R^2^ values were high in our study. The lack of full-length X-ray was also the main reason why only 107 TKAs during a 4-year period were selected. Therefore, although the prediction of our system based solely on the alignments is good, it is necessary to further establish a scoring model based on preoperative function, baseline characteristics, soft-tissue balance status, implant types, and so forth.

## Conclusions

This study developed the first validated, evidence-based scoring system based on lower-extremity angles of alignment to predict early clinical outcomes in TKA and then objectively evaluate the quality of surgery. This new scoring system is precise, practical, convenient, and detailed. We hope it will help surgeons offer timely feedback to patients in clinical practice, provide a quantitative evaluation of TKA quality, and accelerate the progress of scientific research into TKA.

## Data Availability

The data used to support the findings of this study are available for academic using from the corresponding author upon request. To respect participants’ rights to privacy, the information of patients will not be disclosed.
